# High-Intensity Pulsed Electromagnetic Field-Mediated Gene Electrotransfection In Vitro

**DOI:** 10.3390/ijms23179543

**Published:** 2022-08-23

**Authors:** Matej Kranjc, Janja Dermol-Černe, Tjaša Potočnik, Vitalij Novickij, Damijan Miklavčič

**Affiliations:** 1 Faculty of Electrical Engineering, University of Ljubljana, 1000 Ljubljana, Slovenia; 2 Faculty of Electronics, Vilnius Gediminas Technical University, 03227 Vilnius, Lithuania

**Keywords:** electromagnetic field, electroporation, endocytosis, gene transfection, dextran

## Abstract

A high-intensity pulsed electromagnetic field (HI-PEMF) is a non-invasive and non-contact delivery method and may, as such, have an advantage over gene electrotransfer mediated by conventional electroporation using contact electrodes. Due to the limited number of in vitro studies in the field of gene electrotransfection by HI-PEMF, we designed experiments to investigate and demonstrate the feasibility of such a technique for the non-viral delivery of genetic material into cells in vitro. We first showed that HI-PEMF causes DNA adsorption to the membrane, a generally accepted prerequisite step for successful gene electrotransfection. We also showed that HI-PEMF can induce gene electrotransfection as the application of HI-PEMF increased the percentage of GFP-positive cells for two different combinations of pDNA size and concentration. Furthermore, by measuring the uptake of larger molecules, i.e., fluorescently labelled dextrans of three different sizes, we showed endocytosis to be a possible mechanism for introducing large molecules into cells by HI-PEMF.

## 1. Introduction

The introduction of small biomolecules, nucleic acids, proteins, synthetic nanomaterials and drugs into cells is a powerful means of monitoring and deciphering cellular behavior and influencing cellular fate and a cell’s biological functions [1,2,3]. The effects of high-intensity pulsed electromagnetic fields (HI-PEMF) on the permeability of cell membranes have already been observed: in vitro in mammalian cells and microorganisms [4,5] and in vivo in small animals [6,7,8,9]. Various molecules have been used to demonstrate cell membrane permeabilization by HI-PEMF, including propidium iodide [10,11], YO-PRO-1 [10], lucifer yellow [12], cisplatin, bleomycin [6] and calcein [13]. HI-PEMF has been used to enhance viability inhibition [5,14] and disruption of the blood–brain barrier [15]. Recently, HI-PEMF has been used to deliver siRNA molecules to silence enhanced green fluorescent protein (EGFP) [16] in B16F10-EGFP tumors and to deliver plasmid DNA encoding EGFP (pEGFP-N1) in mouse muscle, skin and tumors [9]. Both studies were conducted on animal models in vivo and are one of the few publications on HI-PEMF-mediated gene electrotransfection, along with the initial in vivo study by Kardos and Rabussay [7].

A HI-PEMF proof of concept was first presented in 2012 [12] and is based on time-varying magnetic pulses, which induce an electric field sufficient to trigger cell permeabilization. Due to the high current (≈kA) and voltage (≈kV) requirements, the availability of suitable pulsed power supply systems is limited [11]. The management of the Joule heating [17] and transients adds further complexity to the system design, which is considerably higher compared to conventional electric pulse generators. Most studies with HI-PEMF-induced membrane permeabilization used bipolar pulses. In general, shorter and a greater number of pulses resulted in better membrane permeabilization [11,12]. HI-PEMF is traditionally compared to conventional electroporation or pulsed electric field (PEF) treatment [10,12], which uses high-voltage pulses to increase membrane permeability [18] and is increasingly used in medicine [19], biotechnology [20] and food processing [21]. The use of electroporation as a gene delivery method (gene electrotransfer) was first reported in 1982 [22] and has since reached a broad spectrum of applications in different tissues [23,24]. However, one of the disadvantages of gene electrotransfer that limits the efficiency of transfection is tissue damage associated with the procedure [25,26], especially if the electrical pulse parameters are not chosen appropriately [27]. Another disadvantage is the presence of electrochemical reactions at the electrode–electrolyte tissue interface, which also changes pH and can lead to the denaturation of plasmid DNA [28,29,30] and cell death by electroporation linked with electrolysis [31]. In contrast, HI-PEMF is a non-invasive and non-contact delivery method and may, as such, have an advantage over gene electrotransfer mediated by conventional methods, i.e., contact, electroporation. In a recent in vivo study [9], HI-PEMF treatment was shown as a feasible non-invasive approach to achieve in vivo transfection by enabling the transport of larger molecules such as pDNA encoding EGFP into muscle and skin, while inducing no tissue damage and significantly lower infiltration of inflammatory mononuclear cells compared to conventional electroporation using contact electrodes.

Due to the limited number of in vitro studies in the field of gene electrotransfection induced by HI-PEMF, we designed experiments to investigate the feasibility of such a technique for the non-viral delivery of genetic material into cells in vitro. We focused on the mechanism of internalization of the plasmid by observing the adsorption of the fluorescently labelled plasmid to the plasma membrane, followed by introducing a green fluorescent protein into the cells. We then hypothesized that the HI-PEMF facilitates the uptake of large molecules by endocytosis, which we tested by measuring the uptake of larger molecules, i.e., fluorescently labelled dextrans of three different sizes.

## 2. Results

### 2.1. pDNA Interaction with Cell membrane and Gene Electrotransfection

**Figure 1:**
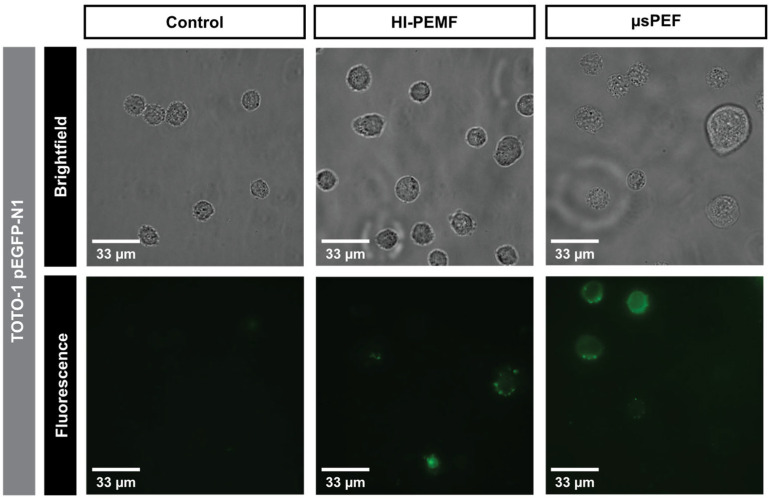
pDNA interaction with cell membrane after the application of HI-PEMF and µsPEF. Images of brightfield and fluorescence taken under the microscope with 500 µg/mL of TOTO-1 labelled pEGFP-N1.

The interaction of pDNA with the cell membrane was visualized after the application of HI-PEMF and µsPEF with TOTO-1 nucleic acid stain. In the absence of pulse delivery, no increase in fluorescence intensity was observed on the level of the cell membrane, indicating no pDNA interaction with the cell membrane, whereas after HI-PEMF and µsPEF, pDNA aggregation was observed at the cell membrane level (Figure 1).

**Figure 2:**
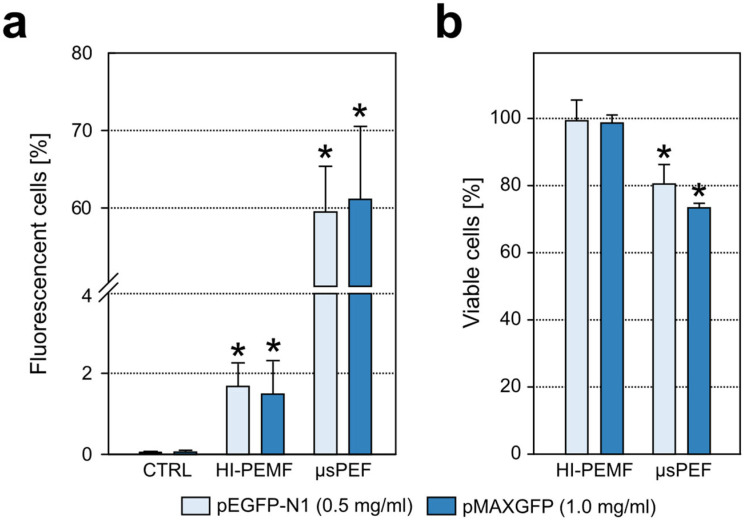
Gene electrotransfection efficiency (**a**) and cell viability (**b**) after the application of HI-PEMF and µsPEF for pEGFP-N1 (0.5 mg/mL) and pMAXGFP (1.0 mg/mL). Asterisk (*) denotes statistical difference between treated sample and its corresponding untreated sample (*p* < 0.05). Mean ± st. deviation is shown for each treatment.

To test whether HI-PEMF can achieve gene electrotransfection, we introduced pDNA coding green fluorescent protein into the cells. We tested two different pDNA sizes: 3.5 kb (pMAXGFP) and 4.7 kb (pEGFP-N1), and two different pDNA concentrations: 0.5 and 1.0 mg/mL. The application of HI-PEMF increased the percentage of GFP-positive cells for two combinations of pDNA size and concentration. With the larger pDNA (pEGFP-N1), we obtained a statistically significant increase in the percentage of GFP-positive cells at 0.5 mg/Ml, as we also did with the smaller pDNA (pMAXGFP) at 1 mg/mL of pDNA concentration (Figure 2a). The application of µsPEF did not increase the percentage of GFP-positive cells for the other two combinations of pDNA size and concentration, i.e., pEGFP-N1 at 1 mg/mL and pMAXGFP at 0.5 mg/mL. As expected, the application of µsPEF increased the percentage of GFP-positive cells for all combinations of pDNA size and concentration. For both combinations of pDNA size and concentration, the application of HI-PEMF did not affect the cell viability. However, cell viability was decreased after the application of µsPEF and was lower compared to HI-PEMF (Figure 2b).

**Figure 3:**
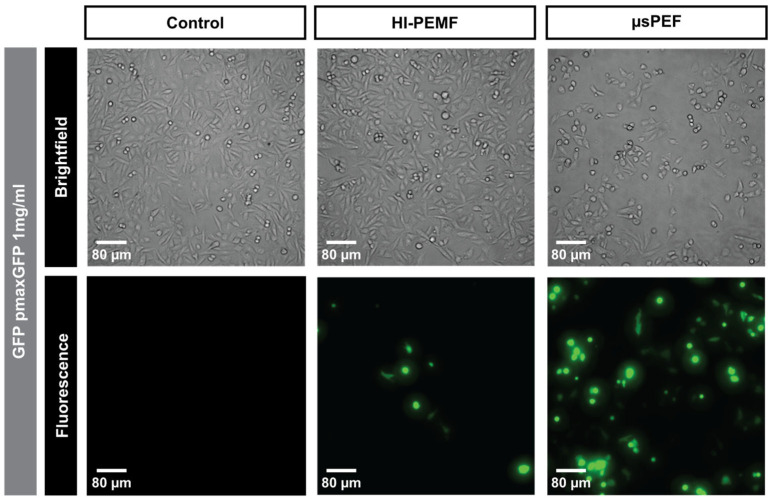
The expression of green fluorescent protein after the application of HI-PEMF and µsPEF.

As shown in Figure 3, the expression of the green fluorescent protein in the cells exposed to HI-PEMF and µsPEF was also visualized under the microscope.

### 2.2. Uptake of Small and Large Molecules

**Figure 4:**
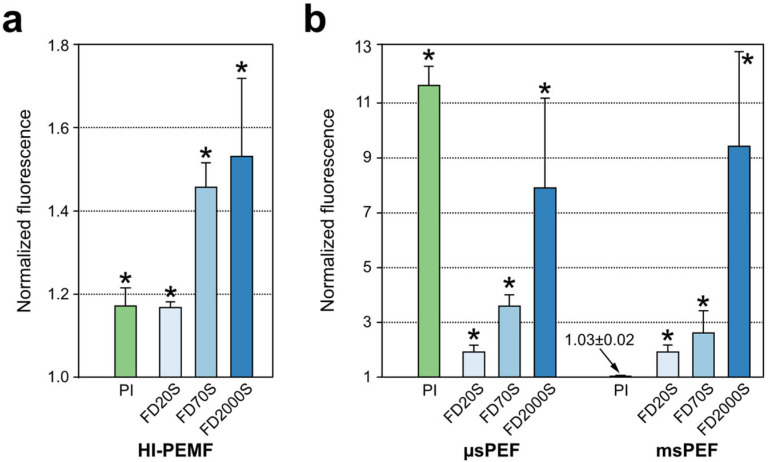
Fluorescence of PI and dextrans for HI-PEMF (**a**) and both PEF pulses (**b**) normalized to corresponding controls—note different ranges in (**a**,**b**) plots. Asterisk (*) denotes statistical difference between treated sample and its corresponding untreated sample (*p* < 0.05). Mean ± standard deviation is shown for each treatment.

The results of the uptake of small (PI) and large (dextrans FD20S, 70S and 2000S) molecules after the application of HI-PEMF pulses and PEF pulses (µsPEF and msPEF) are shown in Figure 4. These results were obtained by flow cytometry and were normalized to the corresponding controls.

**Figure 5:**
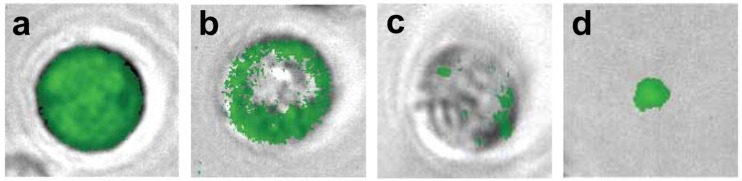
Different distribution of dextrans detected on microscopic images of fluorescently labelled dextrans presented as overlay of brightfield and fluorescence channel: uniform distribution (**a**), confined to cytosol (**b**), clusters (**c**) and debris (**d**).

Treated cells’ suspensions in the presence of dextrans were also imaged under the microscope to confirm the intracellular presence of dextrans. As presented in Figure 5, fluorescent microscopy revealed different intracellular distributions of dextran: uniform distribution (Figure 5a), confined to cytosol (Figure 5b), clusters (Figure 5c) and, as an extracellular formation of dextran, debris (Figure 5d).

**Figure 6:**
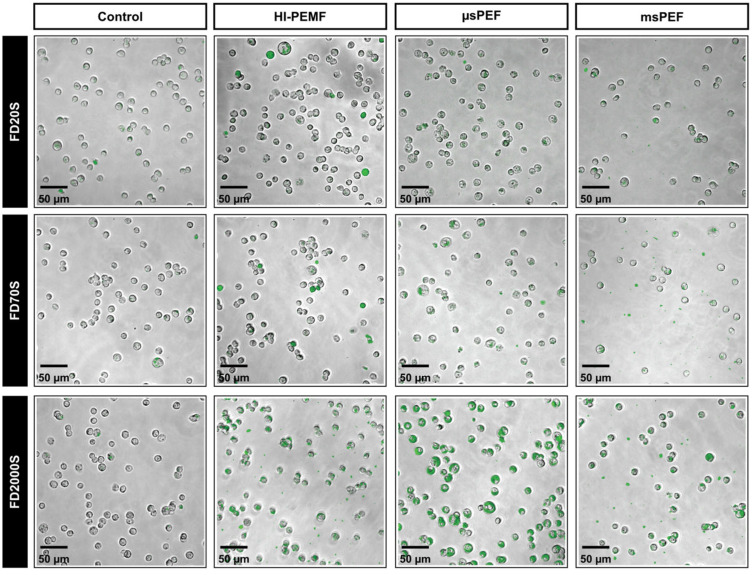
Microscopic images of fluorescently labelled cells with dextrans after application of HI-PEMF, µsPEF and msPEF presented as an overlay of brightfield and fluorescence channel. Each column shows from left to right: control, HI-PEMF, µsPEF and msPEF samples. Each row shows FD20S, FD70S and FD2000S from top to bottom.

As expected, small amounts of dextrans were present in the control samples for all three dextran sizes, mostly in clusters (Figure 6, first column). After treatment with HI-PEMF, FD20S and FD70S, dextrans were either uniformly distributed within cells or they formed clusters within cells, while FD2000S dextran was confined to the cytosol or detected as clusters (Figure 6, second column). Both µsPEF- (Figure 6, third column) and msPEF- (Figure 6, fourth column) treated samples resulted in the dextran in FD20S and FD70S forming clusters in cells. Most of the FD2000S dextran was found confined to the cytosol of cells exposed to µsPEF, while FD2000S formed clusters after exposure to msPEF. Dextrans of all three sizes were also detected as debris after msPEF treatment, while a small number of debris were also found after the HI-PEMF treatment of cells with FD2000S dextran.

## 3. Discussion 

In our study, we investigated the efficiency of gene transfection with HI-PEMF. Since endocytosis is considered to be the most probable pathway of pDNA entering into cells in gene electrotransfer [32], we also investigated the uptake of larger molecules commonly used as markers of endocytosis to test the hypothesis that endocytosis is also involved in HI-PEMF-mediated uptake.

We first showed that HI-PEMF causes DNA adsorption to the membrane (Figure 1), which was for conventional electroporation first reported after the application of longer millisecond and hundreds of microseconds long pulses [33] and, recently, also for short bipolar high frequency microsecond pulses [34]. We also observed that HI-PEMF can induce gene electrotransfection as the application of HI-PEMF increased the percentage of GFP-positive cells for two different combinations of pDNA size and concentration (Figure 2). As shown in Figure 3, the expression of the green fluorescent protein in the cells was also observed under the microscope. However, the percentage of GFP-positive cells was considerably lower than when µsPEF was applied (Figure 2), which can be explained by the much higher applied electric field during µsPEF (at least 80 times) than the electric field induced by HI-PEMF. In addition, non-homogeneous distribution of the induced electric field due to HI-PEMF and the variation in time make the induced electric field due to HI-PEMF substantially different from the electric field established during conventional electroporation using contact electrodes. Therefore, a straightforward comparison between HI-PEMF and conventional electroporation in terms of electric field amplitude can be misleading in the interpretation of results. Nevertheless, despite the lower efficiency, we confirmed, to our knowledge for the first time, successful gene transfer into cells and green fluorescent protein expression by HI-PEMF in vitro. The efficiency of HI-PEMF can be further improved by developing new generators that deliver shorter magnetic pulses [11] and/or by introducing conductive gold nanoparticles that can significantly enhance the permeabilizing effect of HI-PEMF [35].

Endocytosis is a general biological process in which cells take up larger particles or molecules by forming a vesicle and then internalizing it [36]. Endocytosis occurs continuously but can also be enhanced, for example, by the application of long low-voltage electric pulses (far below electroporation pulse parameters) [37,38,39,40], electroporation pulses [41], different mechanochemical stimuli [42], modulated electromagnetic fields [43] and by high-level static magnetic fields in combination with magnetic nanoparticles [44,45]. Endocytosis was also reported as a mechanism of HI-PEMF-mediated uptake of molecules [6,12,46,47], and it has been successfully used to enhance the uptake of molecules using PEF with electric fields whose values are similar to those induced by HI-PEMF [37,38,39]. It is therefore reasonable to assume that high-intensity pulsed electromagnetic fields could also enhance endocytosis. 

To evaluate the uptake of small molecules, we determined the cell uptake of PI (~668 Da) for all pulse protocols. The size of the propidium cation (not the entire propidium molecule) presents a limit on the passage of this dye across the cell membrane, with the longest dimension being 1.5 nm [48]. HI-PEMF caused a small but statistically significant uptake of propidium, as expected and in agreement with previously published reports (Figure 4) [10,11]. µsPEF caused the highest membrane permeabilization of all pulse protocols tested, which is to be expected as these optimized pulse parameters are commonly used to enhance the uptake of similarly sized molecules, e.g., in electrochemotherapy. In contrast, msPEF did not cause membrane permeabilization for PI (Figure 4). One of the plausible reasons for the lack of membrane permeabilization could be related to decreased cell viability due to membrane oxidation and chemical reactions associated with the application of the intense msPEF electrical pulses [49,50]. Similarly, limited cell membrane permeabilization associated with low cell viability of the msPEF protocol was also reported in a recent study that thoroughly evaluated different electric pulse protocols [34]. These results are nevertheless in contradiction with many other reports and need further investigation to understand this apparent discrepancy.

We have also used FITC dextrans to assess the uptake of large molecules, as they are also commonly used as a model molecule for the membrane transport of larger molecules in electroporation studies [41,51,52]. Different sizes of dextrans can be used as approximations for different biologically relevant molecules. For example, FITC dextrans in the range of 20 kDa (FD20s) can be considered to mimic antisense oligonucleotides, in the range of 70 kDa (FD70s), antibodies, and in the range of 2000 kDa (FD2000s), genes [53]. From the flow cytometry data (Figure 4), all three treatments (HI-PEMF, µsPEF and msPEF) achieved significantly higher fluorescence than the control, indicating the adsorption and/or internalization of dextran molecules. The relative increase was greatest with the largest dextran since we used a 5-fold higher concentration of FD2000s than of FD20s or FD70s. Flow cytometry does not distinguish between uptake into the cytosol and possible adsorption to the membrane [38]. Therefore, we quantitatively evaluated the uptake by flow cytometry and then confirmed under the microscope whether the dextrans were internalized or adsorbed on the membrane. In the controls (Figure 6, first column), we detected only a few dextran clusters for all three dextran sizes. Interestingly, no dextran debris (Figure 5d) was found in any of controls, whereas an increased number of debris were present in all msPEF treatments and in the HI-PEMF treatment of cells with FD2000S dextran. As suggested in [52], the debris could be a consequence of the damage caused by the irreversible membrane electroporation or the exchange of molecules between the cytosol and pulsing medium during electroporation, which can significantly disrupt the intracellular microenvironment. Fluorescence microscopy also showed that the intracellular distribution of the delivered dextran after treatment with HI-PEMF varied depending on dextran size (Figure 6, second column). FD20S and FD70S dextrans were found to be uniformly distributed within some of the cells (Figure 5a), while FD2000S dextran was confined to the cytoplasm (Figure 5b). Similar results for the same sizes of dextrans were reported in a study of the ultrasound-induced delivery of macromolecules, in which it was speculated that the intracellular distribution of the molecules was consistent with being limited by the size of passive diffusion through the nuclear pores, suggesting that FD20S and FD70S dextrans were only delivered through the plasma membrane and then distributed in the cell by diffusion [53]. Observation under the microscope also revealed clusters of dextrans (Figure 5c) of all sizes within cells exposed to HI-PEMF, suggesting the uptake of dextran via vesicles by an endocytic process as suggested in the study where authors employed electric fields of values up to 20 V/cm [37], similar to fields induced by HI-PEMF in our present study. The authors in [37] reported that the observed enhanced internalization of macromolecules such as dextran can be partially attributed to a clathrin-dependent pathway, while the rest of the uptake can be attributed to macropinocytotic and clathrin/caveolin-independent pathways or even to a new, yet unknown pathway triggered by low electric fields. Whether similar uptake mechanisms also occur in cells exposed to HI-PEMF needs to be further investigated, e.g., through the introduction of endocytosis inhibitors and endosomal markers [54]. In cells exposed to µsPEF and msPEF treatment, similar dextran clusters were found for all three sizes of dextran (Figure 6), also suggesting an endocytic process, most likely micropinocytosis, as suggested in [41] for electroporation pulses. Similar to the HI-PEMF treatment, but more pronounced in the µsPEF treatment, was the distribution of FD2000S dextran in the cytoplasm of treated cells (Figure 6). In a study of the electroporation-mediated uptake of dextran [52], the authors concluded that convection was probably the dominant transport mode for the cellular uptake of macromolecules, rather than endocytosis, as only few vesicles were observed. Additionally, in [52], the authors focused on the hypothesis that electroporation-mediated transport occurs only through pores, leading to the conclusion that convection is the dominant mechanism. Increased membrane permeability due to oxidative damage to the membrane can also contribute to cell membrane permeability and enable the transport of molecules across the membrane [50]. Interestingly, the largest dextran 2000 kDa is still smaller than the supercoiled plasmid used in our study with a hydrodynamic diameter of about 300 nm [55,56]. Another relevant difference between the uptake of plasmid and dextran is the involvement of electrophoresis, where the plasmid is strongly negatively charged (−2 per base pair, i.e., about −8000) whereas the dextran is only weakly charged (−4 due to the FITC group).

Although the non-viral delivery of genetic material into cells using conventional electroporation is promising, it has some limitations, including the presence of electrochemical reactions of the electrode–electrolyte tissue interface and harmful pH changes around the electrodes [29,30]. The HI-PEMF-induced increase in cell membrane permeability is similar to electroporation, with the important difference that the application of the treatment fields is non-invasive. Even so, both HI-PEMF and conventional electroporation are not fully understood in terms of their mechanisms involved in gene electrotransfer. There are numerous studies on the mechanisms of gene electrotransfer, while the present study is one of the first attempts to unravel the possible mechanisms of HI-PEMF-induced membrane permeabilization and gene transfer.

## 4. Materials and Methods

### 4.1. Pulse Application

**Figure 7:**
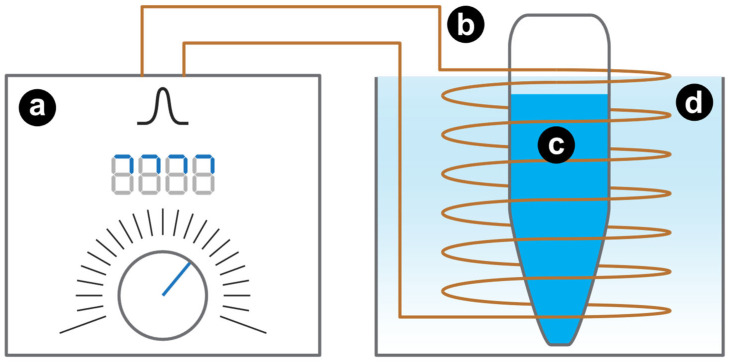
Illustration of the experimental set-up for the application of HI-PEMF in vitro. Custom-made HI-PEMF generator (**a**) was connected to an applicator consisting of a round coil (**b**) with 48 turns. The sterile 0.2 mL PCR standard tube containing the cell suspension (**c**) was placed inside the applicator (**b**). Both the applicator and the PCR tube were immersed in the ice bath (**d**) for the duration of the pulse delivery.

An illustration of the experimental set-up for the application of HI-PEMF in vitro is shown in Figure 7. For the application of pulsed magnetic fields we used custom-made HI-PEMF generator and an applicator consisting of a round coil with 48 turns, both described in detail in [11]. The generator supplied the applicator with unipolar electric pulses that generated a time-varying HI-PEMF in the vicinity of the coil. The inner diameter of the coil was adjusted to match the tip of the standard 0.2 mL sterile PCR tube (ABgene, ThermoFisher Scientific, Waltham, MA, USA), where the cells are placed for the treatment. In the middle of the coil, the applied magnetic field was 6.7 T, while the induced electric field was up to 20 V/cm near the coil windings, linearly declining to 0 towards the center. HI-PEMF samples were treated with most efficient parameters from our previous study [11] (Table 1). During pulse delivery, the HI-PEMF applicator was immersed in ice bath for Joule heating management, thus providing the cooling of the applicator and not exceeding the temperature of 25 °C during treatments.

For the application of conventional electroporation using contact electrodes, we delivered electric pulses to 1 mm electroporation cuvette (VWR, Radnor, PA, USA) using the Electro cell B10 electrical pulse generator (Betatech, Saint-Orens-de-Gameville, France). Since we have recently published a comprehensive study on gene electrotransfer in which we thoroughly evaluated different electric pulse protocols [34], we limited our current evaluation of gene electrotransfer to a single conventional electroporation protocol—µsPEF (Table 1). Still, for the evaluation of endocytosis as an uptake mechanism, we added additional pulse protocol msPEF to HI-PEMF and µsPEF (Table 1).

The maximum treatment volume (40 µL) was limited by the effective volume of the inductor. For each measurement, there was a corresponding negative control that was kept in the ice bath for the duration of the pulse delivery. Immediately after pulse delivery, the samples were transferred into clean 1.5 mL tubes and kept at room temperature until measurement.

### 4.2. Cell Preparation

Chinese Hamster Ovary cells (European Collection of Authenticated Cell Cultures ECACC, cells CHO-K1, cat. no. 85051005, obtained directly from the repository) were grown in 75 cm^2^ culture flasks (Techno Plastic Products AG, Trasadingen, Switzerland) in HAM F-12 growth medium (Cat. no. N6658, Sigma-Aldrich, St. Louis, MO, USA) for 2–3 days in an incubator (Kambič, Semič, Slovenia) at 37 °C and humidified 5% CO_2_. The growth medium (used in this composition through all experiments) was supplemented with 10% fetal bovine serum (cat. no. F9665, Sigma-Aldrich, St. Louis, MO, USA), L-glutamine (cat. no. G7513, Sigma-Aldrich, St. Louis, MO, USA) and antibiotics penicillin/streptomycin and gentamycin (cat. nos. P0781 and G1397, Sigma-Aldrich, St. Louis, MO, USA). On the day of the experiments, cell suspension was prepared by detaching the cells with trypsin-EDTA, diluted 1:9 in Hank’s basal salt solution (StemCell, Vancouver, Canada) and inactivated with the full growth medium. Cells were transferred to a 50 mL centrifuge tube (TPP, Techno Plastic Products AG, Trasadingen, Switzerland) and centrifuged 5 min at 180 g and 4 °C. The supernatant was removed, and cells were resuspended in full growth medium HAM F-12 at cell density 5 × 10^6^ cells/mL.

### 4.3. Plasmids

A 4.7 kb plasmid pEGFP-N1 (Clontech Laboratories Inc, Mountain View, CA, USA) or 3.5 kb pMAXGFP (Lonza, Basel, Switzerland) both encoding green fluorescent protein (GFP) under the control of CMV promotor were used. Plasmid (pDNA) was amplified using Escherichia coli and isolated with HiSpeed Plasmid Maxi Kit (Qiagen, Hilden, Germany). pDNA concentration was spectrophotometrically determined at 260 nm.

### 4.4. pDNA Interaction with the Cell Membrane

To visualize pDNA interaction with the cell membrane TOTO-1 (Molecular Probes, Invitrogen, Eugene, OR, USA) nucleic acid stain was used. Plasmid pEGFP-N1 was labeled with 2.3 × 10^−4^ M TOTO-1 DNA intercalating dye with an average base pair to dye ratio of 5 for 60 min on ice and in the dark. A total of 40 µL of the cells with 500 µg/mL of TOTO-1 labeled pEGFP-N1 were transferred into either a 200 µL PCR tube (ABgene, Portsmouth, NH, USA), followed by the HI-PEMF treatment, or into 2 mm electroporation cuvette (VWR, Radnor, PA, USA), followed by PEF treatment. After treatment, cells were transferred into clean 1.5 mL tube and 1 mL of growth medium was added. Sample was centrifuged for 1 min in tabletop centrifuge then supernatant was removed and cells were resuspended in 1 mL of growth medium. In order to remove the majority of unbound TOTO-1 dye, the washing step was repeated 3 times. Cells were then transferred to LabTek chamber (ThermoFisher Scientific, Waltham, MA, USA) and observed using 100× oil immersion objective (HC FLUOTAR (340) 100 × /1.30, oil, No. 11506368, Leica, Wetzlar, Germany) excited with a 510 nm LED diode (LED8 light source), and emission was measured through DFT51010 filter cube (LED8 filter wheel). Then 16-bit images were acquired with a deep-cooled 4.2 MP sCMOS Leica DFC9000 Gt fast camera in the Leica Application Suite X v3.7.3. (LAS X, Leica, Wetzlar, Germany) software.

### 4.5. Gene Electrotransfection

Gene electrotransfer was evaluated using two different pDNA sizes: 3.5 kb (pMAXGFP) and 4.7 kb (pEGFP-N1), in two different pDNA concentrations: 0.5 and 1.0 mg/mL. For all combinations of pDNA size and concentration, 40 µL of the cells with plasmids were transferred into either a 200 µL PCR tube (ABgene, ThermoFisher Scientific, Waltham, MA, USA), followed by the HI-PEMF treatment, or into 2 mm electroporation cuvette (VWR, Radnor, PA, USA), followed by PEF treatment. After treatment, cells were incubated for 10 min at 37 °C. Afterwards cells were seeded in growth medium into 24-well plate (Techno Plastic Products AG, Trasadingen, Switzerland) for 24 h at 37 °C, 5% CO_2_. After the incubation, cells in 24 wells were trypsinized and resuspended in 200 µL of phosphate buffer saline and percentage of GFP-positive cells was detected using flow cytometer Attune NxT (ThermoFisher Scientific, Waltham, MA, USA) with a blue laser at 488 nm and a 530/30 nm bandpass filter. At every measurement 10,000 events were recorded. Data obtained were analyzed with the Attune NxT software.

For measuring cell survival, 2 × 104 cells were seeded in HAM-F12 growth medium on 96-well plates (Techno Plastic Products AG, Trasadingen, Switzerland) in triplicate. The cells were placed in the humidified incubator (37 C, 5% CO_2_) for 24 h. The cell survival was measured by the -F12 growth medium. Cell survival was determined using the MTS-based Cell Titer 96 AQueous One Solution Cell Proliferation Assay (Promega, USA). Then, 24 h after treatment, 20 µL of MTS reagent was added to each well and the cells were incubated in a humidified incubator (37 C, 5% CO_2_) for a further 2 h. The cells were then allowed to proliferate. Absorbance at a wavelength of 490 nm was measured using a Tecan Infinite M200 spectrophotometer (Tecan, Männedorf, Switzerland). An average absorbance measured in samples containing only HAM-F12 growth medium was subtracted from the absorbance measured in the cell samples. To calculate the percentage of viable cells, the absorbance of each sample was divided by the average absorbance of the control samples.

### 4.6. Dye Loading

We used four different fluorescent dyes, propidium iodide (PI) for evaluation of membrane permeability and three different FITC-labeled dextrans for evaluation of endocytosis. Immediately before treatment, PI was added at a final concentration of 136 µM (Life Technologies, Carlsbad, CA, USA). Three minutes after the treatment, 200 µL of 0.9% NaCl was added to obtain a sufficient volume for the measurements.

FITC-labelled dextrans for the evaluation of endocytosis (fluorescein isothiocyanate dextran FD20S, FD70S and FD2000S, Sigma-Aldrich, St. Louis, MO, USA) were dissolved in distilled water at stock concentrations of 50 µM (FD20S and FD70S) and 10 µM (FD2000S). Immediately before pulse application, FD20S and FD70S were added to the cell suspension at a final concentration of 4.55 µM and FD2000S at a final concentration of 0.91 µM. Samples were centrifuged (1 min, 2000 G, room temperature) 30 min after treatment, supernatant removed, cells resuspended in 200 µL of physiological solution, followed by another centrifugation, removal of supernatant and resuspension in 200 µL of physiological solution, which removed background fluorescence and allowed stable measurements on the flow cytometer and low background fluorescence on the microscopy images.

### 4.7. Flow Cytometry

Flow cytometric analysis was performed on the Attune NxT flow cytometer (Life Technologies, Carlsbad, CA, USA). We measured 10,000 living singlet cells 30 min after the treatment. All samples were excited with a blue laser (488 nm), and the emission of propidium samples was measured through 590/40 nm filter and of the dextran samples through the 530/30 nm filter. We measured the median fluorescence of the living singlet cells. Each point was repeated three times and normalized to the corresponding control.

### 4.8. Fluorescence Microscopy

For microscopy, 50 µL of the resuspended sample was transferred to a well on a 96-well plate, and cells were left to sediment for 30 min before imaging. We used the inverted Thunder Imager Live Cell system, cells were observed through 40× objective (HC PL FLUOTAR L 40 ×/0.60 Corr., no. 11506201, Leica), excited with a 475 nm LED diode (LED8 light source), and emission was measured through DFT51010 filter cube with additional filter at 535/79 nm, (LED8 filter wheel). Then, 16-bit images were acquired with a deep-cooled 4.2 MP sCMOS Leica DFC9000 Gt fast camera in the Leica Application Suite X v3.7.3. (LAS X, Leica, Wetzlar, Germany) software.

### 4.9. Statistical Analysis

All data were tested for a normal distribution with the Kruskal–Wallis test followed by Student–Newman–Keuls test to evaluate the differences between the experimental groups. A *p*-value of <0.05 was considered significant. Statistical analysis was performed in SigmaPlot v11 (Systat Software, Chicago, IL, USA).

## 5. Conclusions

We have successfully demonstrated for the first time that high-intensity pulsed electromagnetic fields induce gene electrotransfection in vitro by introducing a plasmid encoding green fluorescent protein into cells. Even though the effectiveness of electrotransfection induced by HI-PEMF was significantly lower compared to conventional electroporation using contact electrodes, the HI-PEMF approach could have an advantage over conventional electroporation since the HI-PEMF is a non-invasive, contactless and potentially painless gene delivery method. Furthermore, by measuring the uptake of larger molecules, i.e., fluorescently labelled dextrans, we show endocytosis as a possible mechanism for the transfer of molecules mediated by high-intensity pulsed electromagnetic fields.

## Figures and Tables

**Table 1:** Parameters of the pulse protocols used in our study. HI-PEMF and µsPEF treatments were used to assess gene electrotransfer, while msPEF was added along with HI-PEMF and µsPEF to assess endocytosis.

Name of the Treatment	Electric Field (V/cm)	Magnetic Field (T)	Duration of Pulses (µs)	Number of Pulses	Repetition Frequency (Hz)
HI-PEMF	≤20	6.7	20	350	1
µsPEF	1600	/	100	8	1
msPEF	500	/	5000	8	1

## Data Availability

The data presented in this study are available on request from the corresponding author.
